# Higher Intake of Vegetable Protein and Lower Intake of Animal Fats Reduce the Incidence of Diabetes in Non-Drinking Males: A Prospective Epidemiological Analysis of the Shika Study

**DOI:** 10.3390/nu15041040

**Published:** 2023-02-19

**Authors:** Aya Ogawa, Hiromasa Tsujiguchi, Masaharu Nakamura, Koichi Hayashi, Akinori Hara, Keita Suzuki, Sakae Miyagi, Takayuki Kannon, Chie Takazawa, Jiaye Zhao, Yasuhiro Kambayashi, Yukari Shimizu, Aki Shibata, Tadashi Konoshita, Fumihiko Suzuki, Hirohito Tsuboi, Atsushi Tajima, Hiroyuki Nakamura

**Affiliations:** 1 Department of Public Health, Graduate School of Advanced Preventive Medical Sciences, Kanazawa University, 13-1 Takaramachi, Kanazawa 920-8640, Ishikawa, Japan; 2 Faculty of Nutrition, Osaka Seikei College, 3-10-62 Aikawa, Higashiyodogawa-ku, Osaka 533-0007, Osaka, Japan; 3 Department of Hygiene and Public Health, Faculty of Medicine, Institute of Medical, Pharmaceutical and Health Sciences, Kanazawa University, 13-1 Takaramachi, Kanazawa 920-8640, Ishikawa, Japan; 4 Advanced Preventive Medical Sciences Research Center, Kanazawa University, 13-1 Takaramachi, Kanazawa 920-8640, Ishikawa, Japan; 5 Department of Food Sciences and Nutrition, School of Food Sciences and Nutrition, Mukogawa Women’s University, 6-46 Ikebirakicho, Nishinomiya 663-8558, Hyogo, Japan; 6 Innovative Clinical Research Center, Kanazawa University, 13-1 Takaramachi, Kanazawa 920-8640, Ishikawa, Japan; 7 Department of Biomedical Data Science, School of Medicine, Fujita Health University, 1-98 Dengakugakubo, Kutsukake-cho, Toyoake 470-1192, Aichi, Japan; 8 Department of Public Health, Faculty of Veterinary Medicine, Okayama University of Science, 1-3 Ikoi-no-oka, Imabari 794-8555, Ehime, Japan; 9 Department of Nursing, Faculty of Health Sciences, Komatsu University, 14-1 He Mukai-Motoori-Machi, Komatsu 923-0961, Ishikawa, Japan; 10 Third Department of Internal Medicine, University of Fukui Faculty of Medical Sciences, 23-3 Matsuokashimoaiduki, Eiheiji 910-1104, Fukui, Japan; 11 Community Medicine Support Dentistry, Ohu University Hospital, 31-1 Misumidou, Tomitamachi, Kohriyama 963-8611, Fukushima, Japan; 12 Graduate School of Human Nursing, The University of Shiga Prefecture, 2500 Hassaka-cho, Hikone 522-8533, Shiga, Japan; 13 Department of Bioinformatics and Genomics, Graduate School of Advanced Preventive Medical Sciences, Kanazawa University, 13-1 Takaramachi, Kanazawa 920-8640, Ishikawa, Japan

**Keywords:** diabetes, vegetable protein, animal fat, drinking

## Abstract

Although nutrient intake and alcohol consumption are both closely associated with the incidence of diabetes, their interrelationships remain unclear. Therefore, we herein have investigated the interrelationships among nutrient intake, alcohol consumption, and the incidence of diabetes using longitudinal data. This study included 969 residents ≥40 years living in Japan. In 2011 and 2012, a baseline study was conducted using questionnaires on basic demographics, diabetes, nutrient intake, and lifestyle habits. In 2018 and 2019, a follow-up study was performed using questionnaires and medical records on diabetes. Two-way analysis of covariance (two-way ANCOVA) was used to test the interactions of drinking habits and diabetes incidence on nutrients intake. The prospective relationship between nutrient intake at baseline and the incidence of diabetes in the follow-up stratified by drinkers and non-drinkers was evaluated using multiple logistic regression analysis. Interactions were observed for vegetable protein intake (*p* = 0.023) and animal fat intake (*p* = 0.016) in males. Vegetable protein intake negatively correlated with the incidence of diabetes in non-drinkers (odds ratio (OR): 0.208; 95% confidence interval (95% CI): 0.046–0.935; *p* = 0.041). Furthermore, animal fat intake positively correlated with the incidence of diabetes in non-drinkers (OR: 1.625; 95% CI: 1.020–2.589; *p* = 0.041). Therefore, vegetable protein and animal fat intakes in combination with drinking habits need to be considered for the prevention of diabetes.

## 1. Introduction

The prevalence of type 2 diabetes (DM2) is rapidly increasing worldwide and is estimated to reach 592 million individuals by 2035 [1]. According to the National Health and Nutrition Survey reported by the Ministry of Health, Labour and Welfare, Japan, in 2019, 19.7% of Japanese males and 10.8% of Japanese females were suspected to have diabetes [2]. Over the past decade, there has been an increasing trend of a higher proportion in the elderly group. There is a concern that the rapid aging of the Japanese population might lead to a further increase in the prevalence of diabetes. Insulin secretion is lower and also declines at an earlier age in East Asians than in Caucasians in European countries [3]. Furthermore, overnutrition, physical inactivity, obesity, smoking, and heredity factors reduce insulin sensitivity and increase insulin requirements for blood glucose regulation due to insulin resistance. If this condition continues, insulin secretion declines, leading to the development of DM2. Previous studies on the relationship between nutrient intake and diabetes demonstrated that the intake of polyunsaturated fatty acids (PUFAs) [4], dietary fiber [5,6], zinc [7,8,9], magnesium [10], vitamin D [11], and vitamin E [12,13] reduced the risk of diabetes. The nutritional epidemiology of diabetes in the Japanese population is important [14], as their diets differ from those of western countries [15]. In this regard, studies of the Japanese population revealed diets with fiber [16] and magnesium [17] intakes were associated with a reduced risk of DM2 and carbohydrate [18] was associated with an increased risk. Recent studies that focused on the relationship between dietary protein intake and the risk of DM2 reported that the development of DM2 inversely correlated with vegetable protein intake [19,20,21,22], while others found no relationship [23]. The key nutrients associated with the risk of DM2 are fats and carbohydrates, and, thus, the World Health Organization (WHO) recommends controlling the intake of saturated fatty acids (SFAs) and animal fats to prevent DM2 [24]. The Prevention with a Mediterranean Diet (PREDIMED) study, a prospective cohort analysis of the relationship between dietary fat intake and the risk of DM2, showed that the intake of animal fat was associated with a higher risk of DM2 [25], whereas other studies did not [26]. Similarly, the intake of SFAs was related to the risk of DM2 in some studies [27] but not in others [28,29].

Therefore, the relationship between nutrient intake and the risk of diabetes remains unclear. This may be attributed to the involvement of other factors such as lifestyle habits, including alcohol consumption, which may affect nutrient intake and diabetes. Since alcohol produces more than twice as much energy as the same amount of carbohydrates or protein, excessive alcohol consumption has been proposed as a risk factor for diabetes [24,25]. Many studies have shown that alcohol consumption increases the risk of DM2 [25]. However, it has not yet been established whether alcohol consumption increases or decreases the risk of diabetes in relation to the involvement of other nutrients.

Although nutrient intake and alcohol consumption are both considered to be closely associated with the incidence of diabetes, this interrelationship has not yet been examined in detail, particularly using longitudinal data.

Therefore, the present study investigated the interrelationships among nutrient intake—including protein, fat, and carbohydrates—alcohol consumption, and the incidence of diabetes, and compared prospective relationships between nutrient intake and the incidence of diabetes between non-drinkers and drinkers using longitudinal data from a general population in Japan.

## 2. Materials and Methods

### 2.1. Study Design and Subjects

The Shika study, which examines the health status of community-dwelling residents living in Shika town, has been conducted since 2011. The town is located in the center of the Noto Peninsula in Ishikawa Prefecture, Japan, and had a total population of 23,208 in July 2011. The present longitudinal study was conducted utilizing data from the Shika study.

The target population of the present study was residents ≥40 years living in model districts in the town. In 2011 and 2012, a baseline study was conducted using questionnaires on basic demographics, diabetes, nutrient intake, and lifestyle habits. In 2018 and 2019, a follow-up study was performed using questionnaires and medical records from hospitals on diabetes.

Among 2264 eligible residents in the baseline study, 1948 were analyzed (316 non-respondents; response rate of 86.0%). Of these, 1670 individuals were eligible for the follow-up study after the exclusion of 200 deaths, 35 relocations in the town, and 43 moving out of the town. Following the exclusion of 554 individuals who did not respond to the follow-up study or had no medical information (follow-up rate of 66.8%), 1116 individuals were eligible. A total of 969 individuals were ultimately included in analyses after the exclusion of 46 individuals with diabetes at baseline, 10 with an over- or under-reported nutritional intake, and 91 with missing data.

The present study was conducted with the approval of the Ethics Committee of Kanazawa University (No. 1491). Written informed consent was obtained from all participants prior to data collection.

### 2.2. Assessment of Diabetes Incidence

Regarding diabetes in the baseline study, participants were asked if they were currently being treated for diabetes. Concerning the incidence of diabetes in the follow-up period, medical record information on diabetes was collected thorough a medical record collecting system, which we constructed in four hospitals in the study area. In parallel with the system, we used questionnaires asking whether participants had been diagnosed with diabetes by a physician.

### 2.3. Assessment of Nutrient Intake

Nutrient intake was estimated using the brief self-administered dietary history questionnaire (BDHQ). The BDHQ provides information on the average daily frequency of consumption of 58 food and beverage items in the past month. These 58 items were selected from foods or drinks commonly consumed in Japan, mostly based on the food list of the Japanese National Health and Nutrition Survey [30,31]. The questionnaire listed items on all forms of food preparation, including raw, cooked, processed, and pickled foods. In addition, seasonings and cooking oils consumed were included. For eating and drinking habits, the intake of noodle soup and the intensity of seasoning were evaluated. The reported intakes of food and beverages were converted into energy, macronutrient, and micronutrient values using a computer algorithm for BDHQ. Nutrient intake was reported in terms of energy density. Drinking habits were calculated as the average daily net alcohol intake in the past month and classified into two groups: non-drinkers (0 g/day of net alcohol) and drinkers (>0 g/day of net alcohol). The reproducibility and validity of the BDHQ has been already demonstrated [30,31]. Details on the BDHQ are described elsewhere [30,31]. Participants with a reported nutritional intake of less than 600 kcal or more than 4000 kcal energy per day were excluded from the analyses as they were considered to be under- or over-reporters.

### 2.4. Basic Demographics

Data on sex, age, height, weight, smoking, drinking habits, leisure-time physical activities, and the diagnosis of hypertension were obtained using questionnaires. Body mass index (BMI) was calculated by dividing weight (kg) by height squared (m^2^). Smoking was categorized into two groups: current non-smokers (non-smokers and ex-smokers) and current smokers. Regarding leisure-time physical activities, we used the following question: “How many times a week do you exercise?”. The response options included: (1) daily; (2) 5–6 days a week; (3) 3–4 days a week; (4) 1–2 days a week; (5) none.

### 2.5. Statistical Analysis

All analyses were stratified by sex. Between males and females, unpaired Student’s *t*-tests were used for comparisons of the means of continuous variables and chi-square tests for comparisons of the percentages of categorical variables. Regarding basic characteristics in the diabetes-incidence and non-diabetes-incidence groups, unpaired Student’s t-tests were used for comparisons of the means of continuous variables and chi-square tests for comparisons of the proportions of categorical variables. In comparisons of mean nutrient intake between the diabetes and non-diabetes incidence groups, a one-way analysis of covariance (ANCOVA), adjusted for age, BMI, leisure-time physical activities, hypertension, magnesium, and alpha-tocopherol, was used. Two-way ANCOVA was used to test the interactions of drinking habits and diabetes incidence on nutrients intake. Age, BMI, leisure-time physical activities, hypertension, magnesium, and α-tocopherol were used as covariates. The Bonferroni post hoc test was used for nutrients that showed interactions to test differences in nutrient intake between the diabetes and non-diabetes incidence groups stratified by drinkers and non-drinkers. The prospective relationship between nutrient intake and the incidence of diabetes stratified by drinkers and non-drinkers was examined using multiple logistic regression analysis. The covariates were age, BMI, leisure-time physical activities, hypertension, magnesium, and alpha-tocopherol, and each nutrient was entered separately for each analysis.

Statistical Package for Social Science (SPSS, IBM Corp., Tokyo, Japan) version 27 was used for these analyses. The significance of differences was set at *p* < 0.05 for all analyses.

## 3. Results

### 3.1. Participant Characteristics

Table 1 shows participants’ characteristics. The mean age of male subjects was 60.04 ± 10.80 years, whereas that of female subjects was 61.72 ± 11.69 years, with a significant difference (*p* = 0.021). BMI was significantly higher in males than in females (*p* < 0.001). The percentages of subjects who were smokers (*p* < 0.001), drinkers (*p* < 0.001), and with frequent leisure-time physical activities (*p* = 0.047) were significantly higher in males than in females. No significant differences were observed in the incidence of diabetes in the follow-up study between males and females, although it was slightly higher in males (*p* = 0.053). The prevalence of hypertension did not significantly differ between males and females. Regarding comparisons of nutrient intake, the intakes of animal protein (*p* < 0.001), vegetable protein (*p* < 0.001), animal fat (*p* < 0.001), vegetable fat (*p* < 0.001), and carbohydrates (*p* < 0.001) were significantly higher in females than in males.

### 3.2. Comparison of Subjects with and without Diabetes Incidence

Table 2 shows comparisons of subjects with and without diabetes incidence stratified by sex. In males, age was significantly higher in the diabetes incidence group than in the non-diabetes incidence groups (*p* = 0.035), while no significant differences were observed in females. In males, BMI was slightly higher in the diabetes incidence group than in the non-diabetes incidence group (*p* = 0.092), while no significant differences were noted in females. The percentages of smokers, drinkers, those with frequent leisure-time physical activities, and the prevalence of hypertension did not significantly differ between the diabetes and non-diabetes incidence groups in both sexes. Furthermore, mean nutrient intake did not significantly differ between the diabetes and non-diabetes incidence groups in both sexes.

### 3.3. Interactions of Drinking Habits and Diabetes Incidence on Nutrients Intake

Table 3 shows the interactions of drinking habits and diabetes incidence on nutrients intake. Mean nutrient intake did not significantly differ between the diabetes and non-diabetes incidence groups, and no main effect was found. The main effect of mean nutrient intake by drinkers and non-drinkers was noted for carbohydrate intake (*p* = 0.003) in males. Interactions of diabetes incidence and drinking habits on vegetable protein (*p* = 0.023) and animal fat intake (*p* = 0.016) were found in males.

The results of Bonferroni post hoc tests for the non-drinking group showed that the intake of vegetable protein was significantly lower in the diabetes incidence group than in the non-diabetes incidence group (*p* = 0.040). In contrast, no significant differences were observed between the two groups for the drinking group.

In the non-drinking group, the intake of animal fats was significantly higher (*p* = 0.048) in the diabetes incidence group than in the non-diabetes incidence group, while no significant differences were observed between the two groups for the drinking group.

### 3.4. Prospective Relationship between Nutrient Intake and Diabetes Incidence According to Drinking Habits

Table 4 shows the prospective relationship between nutrient intake and the incidence of diabetes according to drinking habits. A negative correlation was observed between vegetable protein intake and the incidence of diabetes in the non-drinking group (odds ratio (OR): 0.208; 95% confidence interval (95% CI): 0.046–0.935; *p* = 0.041). Likewise, a positive correlation was noted between animal fat intake and the incidence of diabetes in the non-drinking group (OR: 1.625; 95% CI: 1.020–2.589; *p* = 0.041).

In other words, the incidence of diabetes was found to be frequent when vegetable protein intake was lower and animal fat intake was higher only in non-drinkers.

## 4. Discussion

The present study examined the interrelationships among nutrient intake, alcohol intake, and the incidence of diabetes utilizing longitudinal data from a general population of middle-aged and elderly Japanese individuals. We also compared the prospective relationship between nutrient intake and the incidence of diabetes between non-drinkers and drinkers. The results obtained showed an interaction of drinking habits and the incidence of diabetes on nutrient intake, demonstrating the preventive effects of vegetable protein intake and the promotive effects of animal fat on the incidence of diabetes in male non-drinkers.

Previous studies reported that a higher intake of vegetable protein was associated with a reduced risk of DM2 [19,20]. A longitudinal study in USA, which followed 165,080 female nurses and 40,722 male health professionals for 22 years, revealed a slight reduction in the risk of diabetes with a higher vegetable protein intake [19].

A cohort study that followed 21,523 people in Australia for approximately 12 years and a meta-analysis integrating 11 cohort studies found an inverse relationship between vegetable protein intake and the development of DM2 in females [20,21]. Another cohort study that followed 2332 Finnish males for approximately 20 years showed that replacing 1% of energy from animal protein with that from vegetable protein reduced the risk of DM2 by 18% (95% CI 0, 32) [22]. A 10-year prospective cohort study examining the association between the intake of soya protein and the risk of diabetes in Japan reported that a higher intake of soya protein reduced the risk of diabetes [32]. In contrast, another study reported no relationship between vegetable protein intake and the incidence of DM2 [23]. These discrepancies among studies may be attributed to racial differences, differences in research methods, or bias caused by subject selection or dropouts, particularly in longitudinal and cohort studies. Bias in the present study was considered to be relatively small because subjects were representative of a community-dwelling population with a high response rate. Furthermore, differences in assessment methods for nutrient intake markedly affect the data obtained. Various types of dietary assessment methods are currently available, with dietary record methods, food frequency questionnaires (FFQ), and dietary history methods mainly used. Due to the burden on participants, dietary record methods are generally only recorded for a few days, which increases the difficulty associated with estimating habitual intake over a long period of time. It is challenging to quantify the actual amount of nutrients consumed by FFQ because information is only available for the limited number of foods listed, and questions are based on food units (ingredients before cooking) rather than menu items. In the present study, we used the BDHQ, which consists of approximately 80 questions and calculates the intake of 58 foods and more than 100 nutrients. Additionally, the BDHQ asks about the meals eaten in the past month and gathers qualitative and quantitative information on an individual’s cooking and seasoning habits, thereby providing a more realistic picture of their eating habits. Even though the BDHQ is not a direct assessment method, certain dietary habits may be assessed in detail with accuracy. Therefore, vegetable protein intake appears to reduce the risk of diabetes incidence, as shown in the present study. More importantly, the present study categorized subjects as non-drinkers and drinkers and found that a higher intake of vegetable protein reduced the risk of diabetes incidence in male non-drinkers only.

Numerous studies have reported a relationship between alcohol consumption and the incidence of diabetes. Since alcohol produces more than twice as much energy as the same weight of carbohydrates or protein, excessive alcohol consumption has been proposed as a risk factor for diabetes [33,34]. However, moderate alcohol consumption has been shown to exert protective effects against the development of DM2 and had no adverse effects on insulin sensitivity in several studies [35,36]. Therefore, it remains unclear whether alcohol consumption increases or decreases the risk of DM2. Given this controversial relationship between alcohol consumption and diabetes, the relationship between vegetable protein intake and the incidence of diabetes needs to be examined in subjects stratified by alcohol consumption, as in the present study. To the best of our knowledge, this is the first study to investigate the interactive effects of alcohol consumption and nutrient intake on the incidence of diabetes. This may support the validity of the present results showing the preventive effects of vegetable protein intake on the development of diabetes incidence in non-drinkers. The results obtained on non-drinkers are consistent with the aforementioned studies showing the preventive effects of vegetable protein against the development of diabetes incidence [19,22], while those on drinkers are in agreement with the findings of studies showing no such effects [23].

Animal fats mainly contain SFAs. The PREDIMED study, a prospective cohort study that followed 3349 Spanish individuals for 3 to 4 years, reported that higher intakes of animal fats and SFAs were associated with an increased risk of DM2 [25]. In addition, a European prospective study that followed 23,631 participants for 3 to 7 years revealed that increased PUFAs/SFAs ratio was associated with a reduced risk of diabetes, regardless of age, sex, a family history of diabetes, and other lifestyle factors [27]. Moreover, a cohort study in the Netherlands and Finland showed that a higher SFAs intake contributed to the risk of impaired glucose tolerance and non-insulin-dependent DM utilizing 20-year follow-up data on 1260 individuals [37]. On the contrary, a study of the Japanese population reported that higher animal fat intake was inversely associated with diabetes [18]. However, a systematic review and meta-analysis of observational studies found no relationship between animal fat intake and the incidence of DM2, whereas diets containing vegetable fat, but not animal fat, were beneficial for the prevention of DM2 [26]. Similarly, a meta-analysis of cohort studies reported no relationship between SFAs intake and the development of DM2 [28,29,38].

The present results demonstrated the interactions of drinking habits and animal fat intake with the incidence of diabetes, and a logistic regression analysis stratifying drinkers and non-drinkers showed that a low animal fat intake was protective against the development of diabetes in male non-drinkers only. By taking drinking habits into consideration as a third factor, our results were consistent with the former studies for non-drinkers and with the latter studies for drinkers. Almost the same discussion as to the association between vegetable protein intake and diabetes incidence can be applied for animal fat intake.

The lack of similar results for vegetable protein and animal fat intakes in females may be due to the small number of female drinkers examined in the present study.

The mechanisms by which vegetable protein, animal fat, and alcohol intake affect the development of diabetes remain unclear. Previous findings have suggested that the amino acid composition of vegetable protein increased the efficiency of amino acid metabolism, thereby reducing the risk of DM2 [39]. However, drinkers consume amino acids when taking alcohol, which may decrease the efficiency of amino acid metabolism. It is possible that a combination of higher vegetable protein intake and non-drinking habits increases the efficiency of amino acids metabolism, thereby decreasing the risk of diabetes incidence.

Several mechanisms have been proposed for the increased risk of diabetes with a high animal fat intake. The fatty acid composition of structural membrane lipids may affect insulin sensitivity. For example, the higher SFAs content of membrane phospholipids was previously shown to increase insulin resistance and the risk of developing diabetes [40,41,42]. Furthermore, alcohol may increase the SFAs content of structural membrane lipids and change fatty acid compositions, thereby reducing insulin sensitivity and consequently increasing insulin resistance. Therefore, a lower intake of animal fats in non-drinkers may effectively reduce the SFAs content of membrane phospholipids and decrease the risk of diabetes.

Foods containing vegetable protein include beans and their processed products, cereals, and vegetables [43,44]. In addition to reducing alcohol consumption, the active consumption of these foods may contribute to the prevention of diabetes. Animal fat is present in fatty meat, lard, beef fat, bacon, cream, cheese, butter, chocolate, and ice cream [44]. Reducing the intake of these foods in addition to alcohol consumption may be important for preventing the development of diabetes. It may be also beneficial to consume vegetable fats, such as olive oil and sesame oil, as alternatives.

The strengths of the present study include its longitudinal design, which allowed prospective and causal relationships between nutrient intake and the incidence of diabetes to be established. In addition, evaluations of the incidence of diabetes were supplemented by the medical records of hospitals to ensure objectivity. Moreover, the participation rate at baseline was high, which may have excluded selection biases. However, this study had several limitations that need to be addressed. Dietary data were subjective because the intake frequencies of foods or food groups were based on a self-administered questionnaire, which may lead to recall biases. In addition, the calculation of nutrients intake did not account for changes in nutrients composition due to cooking, such as heating. Furthermore, protein data from the nutrient calculations were not analyzed in detail other than vegetable and animal categories. Likewise, we could not use more detailed categorized data for fats because they have not been adequately validated. Moreover, supplements were not included in the calculation, although they were included as a question item. The influence of bioactive substances (e.g., antioxidants), which are abundant in vegetables, was also not examined. Furthermore, for some participants, source information on the incidence of diabetes was only self-reported and not corroborated by medical information. Additionally, the questionnaire for the assessment of diabetes has not been tested for reproducibility or validity, although we utilized medical information whenever possible. Finally, the follow-up rate was low, which may have led to withdrawal biases.

## 5. Conclusions

The present study showed that a higher intake of vegetable protein and a lower intake of animal fat were prospectively associated with a lower incidence of diabetes in male non-drinkers. Considering vegetable protein and animal fat intakes in combination with drinking habits might be important for the prevention of diabetes incidence.

## Figures and Tables

**Table 1:** Participants’ characteristics.

	All (*N* = 969)		Males (*N* = 414)		Females (*N* = 555)		
	Mean (*n*)	SD (%)	Mean (*n*)	SD (%)	Mean (*n*)	SD (%)	*p*-Value *
Age	61.00	11.30	60.04	10.80	61.72	11.69	0.021
BMI, kg/m^2^	23.09	3.20	23.59	3.24	22.72	3.09	<0.001
Drinkers, *n* (%)	522	53.90	341	82.37	181	32.61	<0.001
Physical activities							0.047
Daily	116	12.00	52	12.56	64	11.53	
5–6 times a week	91	9.40	43	10.39	48	8.65	
3–4 times a week	98	10.10	30	7.25	68	12.25	
1–2 times a week	148	15.30	73	17.63	75	13.51	
None	516	53.30	216	52.17	300	54.05	
Diabetes incidence, *n* (%)	44	4.50	25	6.04	19	3.42	0.053
Hypertension, *n* (%)	255	26.30	110	26.57	145	26.13	0.877
Nutrition							
Energy, kcal	1888.94	574.50	2114.71	595.95	1720.52	495.38	<0.001
Animal protein, % energy	8.40	3.20	7.92	3.27	8.75	3.12	<0.001
Vegetable protein, % energy	6.49	1.10	6.18	1.05	6.72	1.00	<0.001
Animal fat, % energy	11.13	3.80	10.35	3.88	11.71	3.69	<0.001
Vegetable fat, % energy	12.55	3.70	11.43	3.50	13.38	3.63	<0.001
Carbohydrates, % energy	55.80	8.30	54.38	8.90	56.86	7.60	<0.001

* Continuous variables were examined by unpaired *t*-tests and categorical variables by χ^2^ tests.

**Table 2:** Comparison of subjects with and without diabetes incidence.

	Males (*N* = 414)					Females (*N* = 555)				
	With DI (*N* = 25)		Without DI (*N* = 389)			With DI (*N* = 19)		Without DI (*N* = 536)		
	Mean (*n*)	SD (%)	Mean (*n*)	SD (%)	*p*-Value *	Mean (*n*)	SD (%)	Mean (*n*)	SD (%)	*p*-Value *
Age	64.08	9.31	59.78	10.85	0.035	63.42	7.88	61.66	11.80	0.358
BMI, kg/m^2^	24.57	2.88	23.53	3.26	0.092	24.04	4.22	22.67	3.03	0.177
Drinkers, *n* (%)	20	80.00	321	82.50	0.749	4	21.10	177	33.00	0.274
Physical activities					0.706					0.978
Daily	3	12.00	49	12.60		2	10.50	62	11.60	
5–6 times a week	3	12.00	40	10.30		1	5.30	47	8.80	
3–4 times a week	0	−	30	7.70		2	10.50	66	12.30	
1–2 times a week	5	20.00	68	17.50		3	15.80	72	13.40	
None	14	56.00	202	51.90		11	57.90	289	53.90	
Hypertension, *n* (%)	9	36.00	101	25.96	0.191	5	26.32	140	26.12	0.582
Nutrition										
Energy, kcal	2208.04	660.07	2108.71	592.02	0.743	1901.98	511.61	1714.09	494.06	0.223
Animal protein, % energy	8.40	3.80	7.89	3.23	0.966	8.70	3.16	8.75	3.12	0.831
Vegetable protein, % energy	6.10	0.91	6.19	1.06	0.912	6.66	1.00	6.72	1.00	0.684
Animal fat, % energy	10.73	4.36	10.32	3.86	0.692	11.65	3.49	11.71	3.70	0.979
Vegetable fat, % energy	11.48	3.40	11.42	3.51	0.135	12.59	3.22	13.41	3.64	0.061
Carbohydrates, % energy	54.48	9.57	54.38	8.87	0.607	58.61	6.98	56.80	7.62	0.305

* Unpaired *t*-test and χ^2^ test for basic characteristics; an analysis of covariance for nutrient intake, with covariates adjusted for age, BMI, physical activities, hypertension, magnesium, and alpha-tocopherol. Abbreviation: DI, diabetes incidence.

**Table 3:** Interactions of drinking habits and diabetes incidence on nutrient intake.

	Males (*N* = 414)								*p*-Value			
	Non-Drinkers (*n* = 73)				Drinkers (*n* = 341)							
	With DI (*n* = 5)		Without DI (*n* = 68)		With DI (*n* = 20)		Without DI (*n* = 321)		DI *	Drinking *	DI × Drinking *	Simple Main Effect ^§^
	Mean (*n*)	SD (%)	Mean (*n*)	SD (%)	Mean (*n*)	SD (%)	Mean (*n*)	SD (%)				
Animal protein, % energy	10.12	4.07	7.85	3.39	7.97	3.71	7.89	3.20	0.302	0.545	0.091	
Vegetable protein, % energy	6.07	0.61	6.94	0.99	6.11	0.98	6.03	1.01	0.163	0.111	0.023	Non-drinkers: 0.040
												Drinkers: 0.326
Animal fat, % energy	13.74	3.20	10.13	3.48	9.98	4.35	10.36	3.93	0.262	0.130	0.016	Non-drinkers: 0.048
												Drinkers: 0.149
Vegetable fat, % energy	12.20	2.46	12.45	3.71	11.30	3.63	11.21	3.44	0.232	0.151	0.979	
Carbohydrates, % energy	56.09	7.44	60.88	7.58	54.08	10.15	53.00	8.51	0.732	0.003	0.148	

* Two-way analysis of covariance (two-way ANCOVA). Covariates prepared with age, BMI, physical activities, hypertension, magnesium, and alpha-tocopherol. ^§^ Bonferroni post hoc test. Abbreviation: DI, diabetes incidence.

**Table 4:** Prospective relationship between nutrient intake and diabetes incidence according to drinking habits.

		Exp(β)	95% CI (Lower)	95% CI (Upper)	*p*-Value *
Non-drinkers	Vegetable protein	0.208	0.046	0.935	0.041
(*n* = 73)	Animal fat	1.625	1.020	2.589	0.041
Drinkers	Vegetable protein	1.345	0.773	2.342	0.294
(*n* = 341)	Animal fat	0.870	0.735	1.030	0.106

* Multiple logistic regression analysis. Adjusted for age, BMI, physical activities, hypertension, magnesium, and alpha-tocopherol, with each nutrient entered separately. Abbreviation: CI, confidence interval.

## Data Availability

The data presented in this study are available upon request from the corresponding author. The data are not publicly available due to privacy and ethical policies.
